# Deep learning for student engagement analysis in educational psychology

**DOI:** 10.3389/fpsyg.2026.1764399

**Published:** 2026-05-01

**Authors:** MingYang Sun, ShuangYi Feng

**Affiliations:** College of Social Sciences, The Catholic University of Korea, Seoul, Republic of Korea

**Keywords:** agent driven sequential planner, engagement dynamics forecaster, Manifold Constrained Interaction Filter, student engagement prediction, uncertainty propagation regularizer

## Abstract

**Introduction:**

Student engagement is a pivotal element in educational psychology, significantly impacting learning outcomes and academic achievement. Traditional methods for analyzing student engagement often rely on static models that fail to capture the dynamic and multifaceted nature of engagement. This paper presents an innovative deep learning framework, the Engagement Dynamics Forecaster, which is designed to analyze and predict student engagement patterns with greater accuracy and depth.

**Methods:**

The model comprises three integral components: the Manifold Constrained Interaction Filter, the Agent Driven Sequential Planner, and the Uncertainty Propagation Regularizer. These components are specifically engineered to address the complexities of high-dimensional feature spaces, temporal dependencies, and the inherent uncertainty in predicting engagement. The framework further incorporates constrained optimization refinement and agent-based decision scheduling strategies, enhancing both performance and interpretability. By integrating domain-specific insights with cutting-edge deep learning methodologies, the Engagement Dynamics Forecaster offers a comprehensive and adaptive approach to understanding and enhancing student engagement in educational contexts.

**Results and discussion:**

Empirical results underscore the model's efficacy in linking theoretical constructs of engagement with practical applications, thereby providing invaluable tools for educators and researchers in the field of educational psychology. The model's ability to accurately forecast engagement dynamics holds significant promise for advancing educational strategies and interventions, ultimately contributing to improved educational outcomes.

## 1 Introduction

Student engagement analysis in educational psychology is a critical task that has garnered significant attention due to its profound implications for improving educational outcomes and fostering effective learning environments. Understanding student engagement not only enables educators to identify factors that influence learning behaviors but also provides actionable insights to tailor teaching strategies to individual needs (Fredricks and McColskey, 2012). Moreover, analyzing engagement can help predict academic performance, detect early signs of disengagement, and enhance classroom dynamics (Bhardwaj et al., 2021). This task is particularly necessary in the modern educational landscape, where diverse learning modalities and digital platforms have introduced new challenges in assessing engagement (Nuha et al., 2023). By leveraging computational methods, researchers aim to address these challenges and develop scalable, objective, and data-driven approaches to measure and improve student engagement. The importance of this task lies in its ability to bridge the gap between psychological theories of engagement and practical applications in real-world educational settings, ensuring that students receive personalized support to thrive academically and emotionally (Toti et al., 2020).

Initial efforts to analyze student engagement focused on manually defining indicators such as facial expressions and interaction patterns. These early methods relied on expert knowledge to create rule-based systems that could interpret engagement through observable behaviors (Mandia et al., 2024). While these approaches provided valuable insights and were aligned with psychological theories, they faced challenges in scalability and adaptability due to the extensive manual effort required to define rules (Hang et al., 2024). These systems faced challenges in managing the complexity and variability characteristic of real-world educational environments, which constrained their ability to effectively capture the multifaceted nature of student engagement (Tan, 2025).

As the field evolved, researchers began to employ algorithms capable of learning from structured datasets, moving beyond the constraints of rule-based systems. Techniques such as support vector machines and decision trees were utilized to identify patterns in engagement-related data, offering improved scalability and adaptability (Sharma et al., 2022). These methods allowed for the analysis of diverse data types, including eye-tracking metrics and interaction logs, without the need for explicit domain knowledge (Ahmed et al., 2021). However, the effectiveness of these models was often limited by the quality and quantity of available data, and they struggled to capture the hierarchical and contextual relationships necessary for a deeper understanding of engagement (Mahmood et al., 2024).

The introduction of deep learning models has significantly advanced the analysis of student engagement by enabling the extraction of complex patterns from large-scale datasets. Convolutional neural networks (CNNs) and recurrent neural networks (RNNs) have been employed to analyze visual and temporal data, such as facial expressions and speech signals (Gurcan et al., 2023). Pre-trained models, like transformers, have further enhanced the ability to process multimodal data and understand contextual relationships, making them particularly effective in diverse educational settings (Santana et al., 2020). Despite their superior performance, deep learning models require substantial computational resources and large labeled datasets, and their black-box nature poses challenges in interpretability (Soloviev, 2018). To address these issues, ongoing research seeks to combine the strengths of deep learning with improved interpretability and efficiency, ensuring that educators can effectively utilize these tools to enhance student engagement (Sukumaran and Manoharan, 2025).

Based on the limitations of symbolic AI, machine learning, and deep learning methods, we propose a new framework for student engagement analysis that integrates multimodal data processing, domain-specific knowledge, and efficient model architectures. Our approach aims to overcome the scalability and adaptability issues of symbolic AI, the data dependency of machine learning, and the computational demands of deep learning. By leveraging lightweight yet powerful model architectures, we ensure that our method is accessible and deployable in real-world educational settings. We incorporate mechanisms for interpretability, allowing educators to gain actionable insights into engagement patterns and underlying factors. Our framework also emphasizes the importance of generalizability, ensuring that the model performs consistently across diverse learning environments and student populations. This approach not only addresses the limitations of existing methods but also paves the way for more effective and practical solutions to student engagement analysis in educational psychology.

We summarize our contributions as follows:

- We propose a framework that integrates multimodal data processing and domain-specific knowledge to enhance interpretability and efficiency.
- Our method demonstrates high adaptability and generalizability across diverse educational contexts, ensuring robust performance in various scenarios.
- Experimental results show significant improvements in accuracy and computational efficiency compared to existing methods, validating the effectiveness of our approach.

## 2 Related work

### 2.1 Deep learning in educational psychology

Deep learning has emerged as a transformative tool in educational psychology, enabling researchers to analyze complex patterns in student behavior, cognition, and engagement. By leveraging neural networks, deep learning models can process vast amounts of data, including textual, visual, and behavioral inputs, to uncover insights that traditional statistical methods may overlook. In the context of student engagement analysis, deep learning techniques have been applied to predict academic performance, identify disengagement, and personalize learning experiences (Khenkar et al., 2023). Convolutional neural networks have been utilized to analyze facial expressions and body language captured through video recordings, providing real-time assessments of student engagement during lectures. Similarly, recurrent neural networks and long short-term memory networks have been employed to analyze sequential data, such as students' interactions with online learning platforms, to detect patterns indicative of engagement or disengagement (Kaur et al., 2018). Another significant application of deep learning in educational psychology is sentiment analysis, where natural language processing techniques are used to evaluate students' written responses, discussion posts, or feedback. By training models on labeled datasets, researchers can classify text into categories such as positive, neutral, or negative sentiment, offering insights into students' emotional states and attitudes toward learning. Generative models, such as transformers, have been applied to synthesize personalized feedback for students, enhancing their motivation and engagement (Ashwin and Guddeti, 2018). These advancements underscore the potential of deep learning to revolutionize the study of student engagement by providing scalable, data-driven solutions that complement traditional psychological theories. Despite its promise, the application of deep learning in educational psychology faces challenges related to data privacy, interpretability, and ethical considerations (Mohamad Nezami et al., 2019). Ensuring that models are transparent and explainable is critical for gaining trust among educators and stakeholders. Moreover, the integration of deep learning into educational settings requires interdisciplinary collaboration between computer scientists, psychologists, and educators to align technological innovations with pedagogical goals. As research in this area continues to evolve, deep learning is poised to play a pivotal role in advancing our understanding of student engagement and fostering more effective educational practices (Sowmia et al., 2023).

### 2.2 Behavioral data for engagement analysis

Behavioral data serves as a cornerstone for analyzing student engagement, offering insights into how learners interact with educational content, peers, and instructors. In recent years, the proliferation of digital learning environments has enabled the collection of granular behavioral data, such as clickstream logs, time-on-task metrics, and interaction patterns. Deep learning models have been instrumental in processing and interpreting these data, uncovering latent features that correlate with engagement levels. Sequence modeling techniques, such as recurrent neural networks and long short-term memory networks, have been applied to analyze temporal patterns in students' online activities, identifying moments of high or low engagement (Zhang and Wang, 2025). These models can capture dependencies across time, providing a nuanced understanding of how engagement evolves during a learning session. Another approach involves the use of reinforcement learning to model student behavior and engagement. By treating the learning process as a sequential decision-making problem, reinforcement learning algorithms can simulate how students navigate educational content and make choices that impact their engagement. These models can also be used to optimize instructional strategies, recommending interventions that maximize engagement based on observed behavioral patterns. Adaptive learning systems powered by reinforcement learning can dynamically adjust the difficulty level of tasks or provide targeted feedback to sustain students' interest and motivation. Behavioral data analysis also extends to multimodal learning environments, where students interact with content through various channels, such as text, video, and simulations. Deep learning techniques, such as multimodal fusion, integrate data from multiple sources to provide a comprehensive view of student engagement. Combining eye-tracking data with clickstream logs and facial expression analysis can reveal how students allocate their attention and respond to different types of stimuli (Abdulkader et al., 2023). These insights can inform the design of more engaging and effective learning experiences. The use of behavioral data for engagement analysis raises important questions about data privacy and ethical considerations. Ensuring that students' data are collected and analyzed responsibly is paramount, particularly in sensitive contexts such as educational psychology. Researchers must also address issues of bias and fairness in their models, as behavioral data may reflect systemic inequalities or cultural differences. Despite these challenges, the integration of deep learning with behavioral data analysis holds significant promise for advancing our understanding of student engagement and improving educational outcomes (Kamath et al., 2021).

### 2.3 Emotion recognition in learning environments

Emotion recognition has gained significant attention in the study of student engagement, as emotions play a critical role in shaping learning experiences and outcomes. Advances in deep learning have enabled the development of sophisticated models for detecting and analyzing emotions in educational settings, using data from facial expressions, voice tone, and physiological signals (Ikram et al., 2023). Convolutional neural networks are commonly employed for facial emotion recognition, leveraging image data to classify expressions such as happiness, frustration, or boredom. These models can be integrated into classroom environments to provide real-time feedback on students' emotional states, allowing educators to adapt their teaching strategies accordingly. Facial analysis, speech-based emotion recognition has emerged as a powerful tool for understanding student engagement. Deep learning techniques, such as spectrogram-based convolutional neural networks and recurrent architectures, are used to analyze audio features, including pitch, tone, and rhythm, to infer emotional states (Mandia et al., 2023). This approach is particularly useful in online learning environments, where verbal communication may be the primary mode of interaction. By identifying emotions such as enthusiasm or confusion in students' voices, educators can tailor their responses to address individual needs and foster a more engaging learning experience. Physiological data, such as heart rate, skin conductance, and brain activity, also provide valuable insights into students' emotional states. Deep learning models, including autoencoders and long short-term memory networks, have been applied to process these data, identifying patterns that correlate with engagement and emotional arousal. Wearable devices equipped with sensors can monitor students' physiological responses during learning activities, offering a window into their emotional experiences. These data can be combined with other modalities, such as facial and speech analysis, to create a holistic understanding of student engagement. The application of emotion recognition in learning environments is not without challenges (Santoni et al., 2024). Ensuring the accuracy and reliability of emotion detection models is critical, as misinterpretations could lead to inappropriate interventions. Moreover, the use of sensitive data, such as facial images and physiological signals, raises ethical concerns related to privacy and consent. Researchers must navigate these issues carefully, balancing the potential benefits of emotion recognition with the need to protect students' rights and well-being. Despite these challenges, emotion recognition represents a promising avenue for enhancing student engagement analysis, offering insights that can inform personalized and emotionally intelligent educational practices.

Recent studies have explored machine learning and deep learning approaches for automatic student engagement detection from classroom or online learning videos. Machine learning frameworks have been applied to identify engagement levels in classroom environments by analyzing observable behavioral cues extracted from student video recordings (Alruwais and Zakariah, 2023). Ensemble learning methods have also been investigated for engagement level detection and emotion analysis in learning scenarios, showing improved robustness compared with single model approaches (Mazumder et al., 2024). Several works have further explored deep learning techniques for engagement recognition from visual data. Domain adaptation strategies have been used to improve cross domain engagement prediction in classroom videos (Thomas et al., 2022). Affective computing approaches based on convolutional neural networks have been proposed to jointly model emotional states and engagement patterns, demonstrating improved performance in engagement detection tasks (Thiruthuvanathan and Krishnan, 2025). Transformer based video architectures have been applied to engagement estimation tasks. These models are capable of capturing long range spatial and temporal dependencies in classroom videos, enabling more accurate engagement prediction from student behavioral sequences (Tran et al., 2025). These studies highlight the growing interest in leveraging computer vision and deep learning techniques to analyze behavioral engagement in educational environments. Building upon these efforts, the proposed framework focuses on modeling engagement dynamics from temporal behavioral observations by integrating structured feature representation learning and sequential decision modeling.

## 3 Method

### 3.1 Overview

This section delineates the methodological framework for analyzing student engagement within the realm of educational psychology, employing deep learning techniques. The proposed approach is crafted to navigate the complexities inherent in modeling engagement dynamics, which are shaped by intricate interactions among students, educational content, and instructional strategies. We introduce a novel model, the *Engagement Dynamics Forecaster*, designed to capture and predict engagement patterns through a data-driven methodology. The model is fortified by meticulously designed modules and strategies that ensure its robustness and adaptability to the domain-specific challenges posed by educational psychology.

The framework is organized into three principal components, each elaborated in the subsequent subsections. Initially, Section 3.2 provides the necessary preliminaries to formalize the problem of student engagement analysis. This includes defining the mathematical notations and structures utilized throughout the paper, as well as framing the problem in alignment with the objectives of the proposed model. The preliminaries establish a foundation for comprehending the intricate relationships between various factors influencing student engagement, such as temporal dependencies, interaction patterns, and contextual variables. Section 3.3 introduces the *Engagement Dynamics Forecaster*, a deep learning-based model that leverages three pivotal modules: the *Manifold Constrained Interaction Filter*, the *Agent Driven Sequential Planner*, and the *Uncertainty Propagation Regularizer*. Each module is crafted to address specific challenges in modeling engagement. The *Manifold Constrained Interaction Filter* is dedicated to extracting meaningful interaction patterns from high-dimensional data while preserving the underlying manifold structure. The *Agent Driven Sequential Planner* models the sequential decision-making processes of students and educators, capturing the temporal evolution of engagement. The *Uncertainty Propagation Regularizer* enhances the model's robustness by explicitly accounting for uncertainty in predictions, which is crucial in educational psychology where data can be noisy and incomplete. Section 3.4 details the strategies employed to augment the performance and interpretability of the *Engagement Dynamics Forecaster*. A *constrained optimization refinement* approach is proposed to ensure the model adheres to domain-specific constraints, such as maintaining consistency with psychological theories of engagement. An *agent-based decision scheduling* strategy is introduced, leveraging insights from the *Agent Driven Sequential Planner* to optimize instructional interventions and improve student outcomes. These strategies are designed to bridge the gap between theoretical models of engagement and their practical applications in educational settings. The proposed methodology constitutes a comprehensive framework for analyzing and predicting student engagement in educational psychology. By integrating advanced deep learning techniques with domain-specific insights, the *Engagement Dynamics Forecaster* serves as a potent tool for understanding and enhancing the learning experience. The following sections provide a detailed exposition of each component of the framework, highlighting the theoretical underpinnings, technical innovations, and practical implications of the proposed approach.

Student engagement in educational psychology is commonly described as a multidimensional construct that reflects students' involvement in learning activities through behavioral, emotional, and cognitive processes. In this work, engagement is operationalized through observable behavioral indicators that can be extracted from video-based learning observations, including attention patterns, interaction responsiveness, and affect related visual cues. The proposed computational framework is designed to model these engagement related processes through corresponding architectural components. The Manifold Constrained Interaction Filter captures stable representations of observable behavioral signals by projecting raw visual features into a structured latent manifold space. This component focuses on extracting consistent behavioral patterns from high dimensional visual observations, which correspond to measurable indicators of student engagement. The Agent Driven Sequential Planner models the temporal dynamics of engagement by representing the evolution of behavioral states across time. Multiple agents are introduced to capture different aspects of engagement dynamics, including cognitive engagement reflected in sustained attention, emotional engagement reflected in affective expressions, and social engagement reflected in interaction related behavioral cues. Through sequential planning and interaction modeling, the framework learns temporal dependencies among these engagement related factors. The Uncertainty Propagation Regularizer accounts for the inherent variability and ambiguity in behavioral observations of engagement. Since engagement indicators derived from visual signals may be noisy or partially observable, uncertainty estimation is incorporated to stabilize the learning process and improve prediction robustness. Through this design, the proposed framework establishes a structured mapping between psychological engagement constructs and computational modeling components, enabling engagement dynamics to be represented through observable behavioral features and modeled through temporal decision processes.

### 3.2 Preliminaries

This section delineates the problem of student engagement analysis within educational psychology, establishing the mathematical framework and notations essential for the proposed methodology. The aim is to model and predict the dynamics of student engagement over time, utilizing deep learning techniques to capture both temporal and interactional patterns. Let the set of students be denoted as $S=\left\{s_{1},s_{2},\ldots ,s_{N}\right\}$, where *N* represents the total number of students. Each student *s*_*i*_ is associated with a sequence of engagement observations ${\text{E}}_{i}=\left\{{\text{e}}_{i}^{1},{\text{e}}_{i}^{2},\ldots ,{\text{e}}_{i}^{T}\right\}$, with *T* indicating the total number of time steps, and ${\text{e}}_{i}^{t}\in {\mathbb{R}}^{d}$ being a *d*-dimensional feature vector encapsulating engagement-related attributes at time *t*.

To capture the temporal evolution of engagement, a sequence of interaction states ${\text{I}}_{i}=\left\{{\text{i}}_{i}^{1},{\text{i}}_{i}^{2},\ldots ,{\text{i}}_{i}^{T}\right\}$ is defined, where ${\text{i}}_{i}^{t}\in {\mathbb{R}}^{k}$ represents the latent interaction state of student *s*_*i*_ at time *t*. These states are influenced by the student's historical engagement patterns and external factors, including instructional strategies or peer interactions. The relationship between engagement observations and interaction states is formally expressed as:


$$\begin{array}{l} {\text{i}}_{i}^{t}=f_{\text{int}}\left({\text{E}}_{i}^{1:t},{\text{C}}_{i}\right), \end{array}$$ (1)

where *f*_int_(·) maps the historical engagement sequence ${\text{E}}_{i}^{1:t}$ and contextual factors **C**_*i*_ to the latent interaction state ${\text{i}}_{i}^{t}$.

The primary objective is to forecast future engagement levels, denoted as ${\hat{\text{E}}}_{i}^{t+1:t+H}=\left\{{\hat{\text{e}}}_{i}^{t+1},{\hat{\text{e}}}_{i}^{t+2},\ldots ,{\hat{\text{e}}}_{i}^{t+H}\right\}$, where *H* is the prediction horizon. This forecasting task is formulated as:


$$\begin{array}{l} {\hat{\text{E}}}_{i}^{t+1:t+H}=f_{\text{pred}}\left({\text{E}}_{i}^{1:t},{\text{C}}_{i},{\text{I}}_{i}^{1:t}\right), \end{array}$$ (2)

where *f*_pred_(·) utilizes historical engagement data, contextual factors, and latent interaction states to estimate future engagement levels.

To ensure model robustness, manifold constraints are imposed on the interaction states **I**_*i*_. It is assumed that these states lie on a low-dimensional manifold $M\subset {\mathbb{R}}^{k}$, capturing the intrinsic structure of engagement dynamics. This constraint is expressed as:


$$\begin{array}{l} {\text{i}}_{i}^{t}\in M,\;\forall t\in \left\{1,2,\ldots ,T\right\}. \end{array}$$ (3)

The manifold M is characterized by basis functions {ϕ_1_, ϕ_2_, …, ϕ_*m*_}, allowing any interaction state ${\text{i}}_{i}^{t}$ to be approximated as:


$$\begin{array}{l} {\text{i}}_{i}^{t}\approx \overset{m}{\underset{j=1}{\sum }}\alpha_{j}^{t}\phi_{j},\;\alpha_{j}^{t}\in \mathbb{R}, \end{array}$$ (4)

where $\alpha_{j}^{t}$ are the coefficients of the basis functions at time *t*.

An agent-based framework is introduced to model decision-making processes influencing engagement dynamics. Each student is treated as an agent interacting with the learning environment, making sequential decisions to optimize engagement. Let ${\text{a}}_{i}=\left\{{\text{a}}_{i}^{1},{\text{a}}_{i}^{2},\ldots ,{\text{a}}_{i}^{T}\right\}$ denote the sequence of actions taken by student *s*_*i*_, where ${\text{a}}_{i}^{t}\in A$ and A is the action space. The evolution of engagement is described as a function of interaction states and actions:


$$\begin{array}{l} {\text{e}}_{i}^{t+1}=f_{\text{eng}}\left({\text{i}}_{i}^{t},{\text{a}}_{i}^{t}\right), \end{array}$$ (5)

where *f*_eng_(·) models the transition from the current interaction state and action to the next engagement observation.

To account for uncertainty in engagement predictions, a probabilistic framework is incorporated. Let $p\left({\text{E}}_{i}^{t+1:t+H}|{\text{E}}_{i}^{1:t},{\text{C}}_{i},{\text{I}}_{i}^{1:t}\right)$ denote the predictive distribution over future engagement levels. The goal is to estimate this distribution, parameterized as:


$$\begin{array}{l} p\left({\text{E}}_{i}^{t+1:t+H}|{\text{E}}_{i}^{1:t},{\text{C}}_{i},{\text{I}}_{i}^{1:t}\right)=\overset{H}{\underset{h=1}{\prod }}p\left({\text{e}}_{i}^{t+h}|{\text{E}}_{i}^{1:t},{\text{C}}_{i},{\text{I}}_{i}^{1:t}\right), \end{array}$$ (6)

where $p\left({\text{e}}_{i}^{t+h}|\cdot \right)$ represents the conditional distribution of engagement at time *t*+*h*.

The optimization problem is formulated as minimizing the discrepancy between predicted and observed engagement levels while satisfying manifold constraints and incorporating the agent-based decision-making framework. This is expressed as:


$$\begin{array}{l} \underset{\text{Θ}}{\min}L\left(\hat{\text{E}},\text{E}\right)+{\text{λ}}_{1}R_{\text{manifold}}\left(\text{I}\right)+{\text{λ}}_{2}R_{\text{agent}}\left(\text{a}\right), \end{array}$$ (7)

where $L\left(\hat{\text{E}},\text{E}\right)$ is the prediction loss, $R_{\text{manifold}}\left(\text{I}\right)$ enforces manifold constraints, $R_{\text{agent}}\left(\text{a}\right)$ regularizes agent-based actions, and λ_1_, λ_2_ are hyperparameters controlling the trade-off between these terms.

This formalization provides the foundation for the subsequent sections. In Section 3.3, the proposed *Engagement Dynamics Forecaster* is introduced, integrating the *Manifold Constrained Interaction Filter, Agent Driven Sequential Planner*, and *Uncertainty Propagation Regularizer* to address the challenges of student engagement analysis. In Section 3.4, the *constrained optimization refinement* and *agent-based decision scheduling* strategies are detailed to enhance the model's performance and interpretability.

### 3.3 Engagement dynamics forecaster

**Figure 1:**
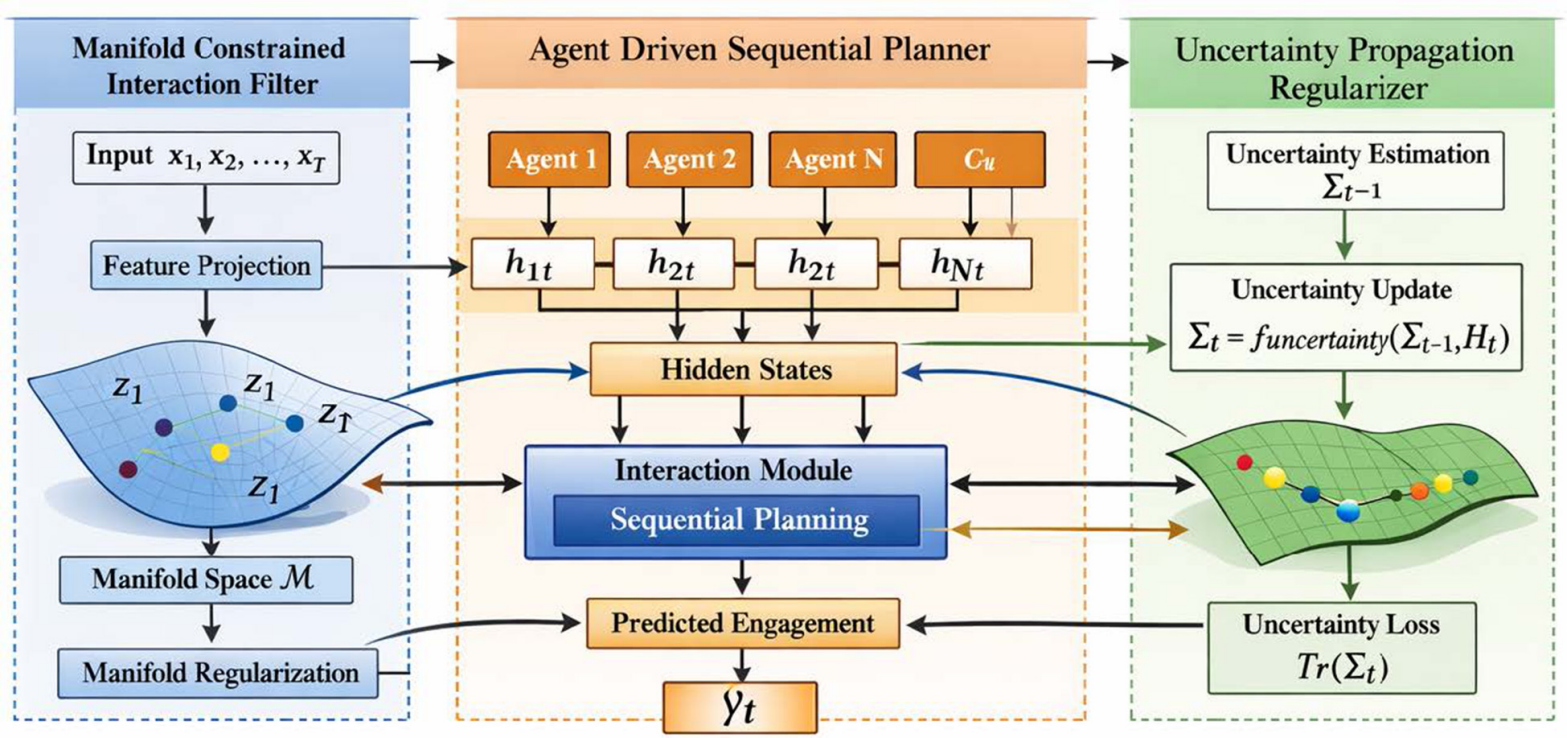
The framework contains three major components: Manifold Constrained Interaction Filter, Agent Driven Sequential Planner, and Uncertainty Propagation Regularizer. The input behavioral sequence is first processed through a feature projection module that maps high dimensional observations into a structured manifold space while enforcing manifold consistency through a regularization mechanism. The learned representations are then passed to a multi agent sequential planning module where multiple agents maintain hidden states and interact through an interaction module to capture temporal engagement dynamics. The planner aggregates agent states to produce engagement predictions over time. An uncertainty propagation module estimates and updates predictive uncertainty during temporal modeling and introduces an uncertainty regularization objective to improve robustness and stability of the predictions. The overall architecture integrates manifold representation learning, multi agent temporal reasoning, and uncertainty aware regularization to model complex engagement dynamics.

The proposed model, termed the *Engagement Dynamics Forecaster*, is designed to capture and predict student engagement patterns in educational psychology through a novel integration of manifold constraints, agent-driven planning, and uncertainty-aware regularization. This section details the architecture and mathematical formulation of the model, emphasizing its unique components: the *Manifold Constrained Interaction Filter*, the *Agent Driven Sequential Planner*, and the *Uncertainty Propagation Regularizer*. The overall architecture of the proposed Engagement Dynamics Forecaster is illustrated in Figure 1. The framework consists of three major components: the Manifold Constrained Interaction Filter, the Agent Driven Sequential Planner, and the Uncertainty Propagation Regularizer. Given a sequence of observed behavioral features extracted from student learning videos, the interaction filter first projects the high dimensional input features into a structured manifold representation. The agent driven sequential planner then models the temporal dynamics of engagement through multiple interacting agents that capture cognitive, emotional, and social aspects of student behavior. The uncertainty propagation module estimates prediction uncertainty and introduces regularization to improve model robustness during training.

The *Engagement Dynamics Forecaster* operates on a sequence of observed student behaviors and contextual features, denoted as **X** = {**x**_1_, **x**_2_, …, **x**_*T*_}, where ${\text{x}}_{t}\in {\mathbb{R}}^{d}$ represents the feature vector at time step *t*, and *T* is the total number of time steps. The goal is to predict a sequence of engagement states **Y** = {**y**_1_, **y**_2_, …, **y**_*T*_}, where ${\text{y}}_{t}\in {\mathbb{R}}^{k}$ corresponds to the engagement state at time *t*, and *k* is the dimensionality of the engagement representation. The model is designed to address the challenges of high-dimensional feature spaces, temporal dependencies, and uncertainty in engagement prediction.

#### 3.3.1 Manifold constrained interaction filter

The first component is responsible for projecting the input features **X** onto a lower-dimensional manifold that preserves the intrinsic structure of the data while filtering out irrelevant noise. Let $M\subset {\mathbb{R}}^{d}$ denote the manifold on which the engagement-related features lie. The projection is achieved through a mapping function $\phi :{\mathbb{R}}^{d}\to M$, parameterized by a neural network. The projected features are given by:


$$\begin{array}{l} {\text{z}}_{t}=\phi \left({\text{x}}_{t}\right),\;{\text{z}}_{t}\in M,\;t=1,\ldots ,T. \end{array}$$ (8)

To ensure that the learned manifold M captures meaningful interactions, we impose a manifold regularization term $R_{\text{manifold}}$ during training, defined as:


$$\begin{array}{l} R_{\text{manifold}}=\overset{T}{\underset{t=1}{\sum }}||{\text{z}}_{t}-{\text{Proj}}_{M}\left({\text{z}}_{t}\right){||}^{2}, \end{array}$$ (9)

where ${\text{Proj}}_{M}$ is the projection operator onto the manifold M.

#### 3.3.2 Agent driven sequential planner

The second component models the temporal dependencies in the engagement states by leveraging a multi-agent framework. Each agent is responsible for a specific aspect of engagement dynamics, and their interactions are governed by a sequential planning mechanism. Let ${\text{h}}_{t}^{i}$ denote the hidden state of the *i*-th agent at time *t*, and ${\text{H}}_{t}=\left[{\text{h}}_{t}^{1},{\text{h}}_{t}^{2},\ldots ,{\text{h}}_{t}^{N}\right]$ represent the collective hidden states of *N* agents. The evolution of these states is modeled as:


$$\begin{array}{l} {\text{h}}_{t}^{i}=f_{\text{agent}}^{i}\left({\text{h}}_{t-1}^{i},{\text{z}}_{t},{\text{H}}_{t-1}\right),\;i=1,\ldots ,N, \end{array}$$ (10)

where $f_{\text{agent}}^{i}$ is the update function for the *i*-th agent, parameterized by a recurrent neural network. The output of the *Agent Driven Sequential Planner* is a set of engagement predictions ${\hat{\text{y}}}_{t}$, computed as:


$$\begin{array}{l} {\hat{\text{y}}}_{t}=g_{\text{planner}}\left({\text{H}}_{t}\right), \end{array}$$ (11)

where *g*_planner_ is a decoding function.

#### 3.3.3 Uncertainty propagation regularizer

The third component addresses the inherent uncertainty in engagement prediction by explicitly modeling the uncertainty in the predicted states. Let **Σ**_*t*_ denote the covariance matrix representing the uncertainty at time *t*. The regularizer encourages the model to propagate uncertainty through the temporal dynamics, ensuring robust predictions. The uncertainty propagation is modeled as:


$$\begin{array}{l} \Sigma_{t}=f_{\text{uncertainty}}\left(\Sigma_{t-1},{\text{H}}_{t}\right), \end{array}$$ (12)

where *f*_uncertainty_ is a learnable function. The regularization term $R_{\text{uncertainty}}$ is defined as:


$$\begin{array}{l} R_{\text{uncertainty}}=\overset{T}{\underset{t=1}{\sum }}\text{Tr}\left(\Sigma_{t}\right), \end{array}$$ (13)

where Tr(·) denotes the trace of a matrix.

The objective function for training the *Engagement Dynamics Forecaster* combines the manifold regularization, uncertainty regularization, and a prediction loss $L_{\text{pred}}$:


$$\begin{array}{l} L=L_{\text{pred}}+{\text{λ}}_{\text{manifold}}R_{\text{manifold}}+{\text{λ}}_{\text{uncertainty}}R_{\text{uncertainty}}, \end{array}$$ (14)

where λ_manifold_ and λ_uncertainty_ are hyperparameters controlling the trade-off between the terms.

### 3.4 Constrained optimization refinement and agent-based decision scheduling

**Figure 2:**
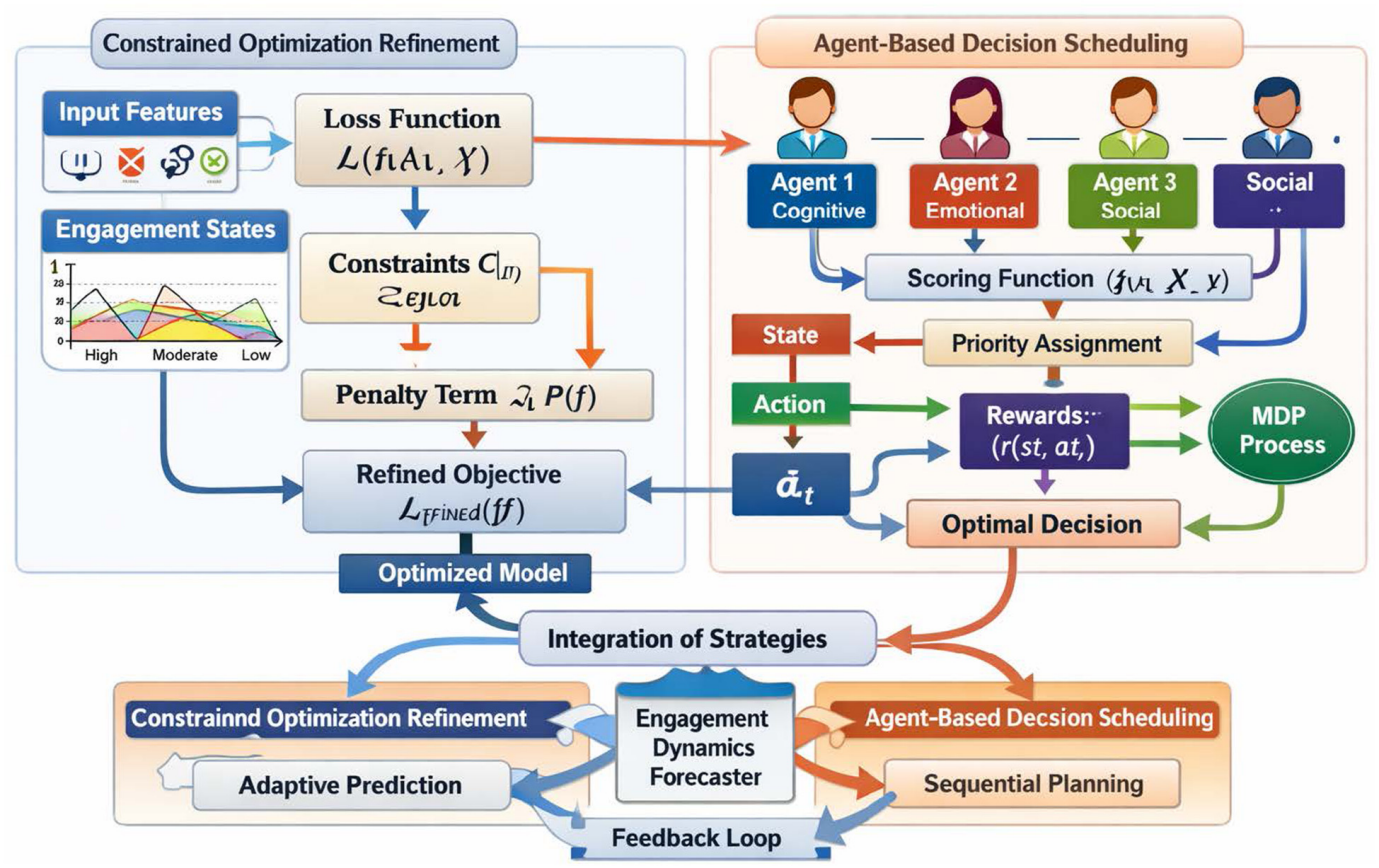
The figure presents the integration of Constrained Optimization Refinement and Agent-Based Decision Scheduling for modeling and predicting student engagement dynamics. On the left side, student interaction features and engagement states are processed through a constrained optimization pipeline including a loss evaluation module, constraint analysis, and penalty adjustment to obtain a refined objective and an optimized predictive model. On the right side, multiple agents representing different engagement factors such as cognitive, emotional, and social aspects are coordinated through a scheduling mechanism that assigns priorities to agents based on the current engagement condition. The sequential decision process follows a reinforcement learning structure with states, actions, rewards, and policy learning. Both strategies are integrated through a feedback loop to form the Engagement Dynamics Forecaster, enabling adaptive prediction, sequential planning, and interpretable analysis of engagement patterns in educational environments.

In this subsection, we elaborate on the strategic innovations that underpin the Engagement Dynamics Forecaster, focusing on the dual mechanisms of constrained optimization refinement and agent-based decision scheduling. These strategies are designed to address the inherent complexities of student engagement analysis in educational psychology, where dynamic interactions and uncertainty play pivotal roles. By leveraging these strategies, the proposed model achieves a robust and adaptive framework for predicting engagement patterns. Figure 2 further illustrates the optimization and decision scheduling process used in the framework. The constrained optimization module refines the learning objective by enforcing domain specific constraints on engagement representations, while the agent based decision scheduling module models engagement evolution as a sequential decision process. These two mechanisms interact through a feedback loop to produce adaptive engagement predictions and improve the stability of the training process.

#### 3.4.1 Constrained optimization refinement

The constrained optimization refinement is central to ensuring that the Engagement Dynamics Forecaster operates within the bounds of meaningful and interpretable solutions. Let X represent the manifold of student interaction features, and Y denote the engagement states. The mapping f:X→Y is constrained by a set of conditions C, which encapsulate domain-specific knowledge and empirical observations. Formally, the optimization problem can be expressed as:


$$\begin{array}{l} \underset{f}{\min}L\left(f\left(X\right),Y\right)\;\text{subject to}\;C\left(f\right)\leq \epsilon , \end{array}$$ (15)

where L is the predictive loss function, and ϵ is a tolerance parameter that governs the permissible deviation from the constraints. The constraints C are designed to capture critical properties such as monotonicity, sparsity, and smoothness in engagement dynamics.

To refine the optimization process, we introduce a penalty term P(f) that quantifies the violation of constraints:


$$\begin{array}{l} L_{\text{refined}}\left(f\right)=L\left(f\left(X\right),Y\right)+\text{λ}P\left(f\right), \end{array}$$ (16)

where λ is a regularization parameter. The penalty term P(f) is defined as:


$$\begin{array}{l} P\left(f\right)=\overset{N}{\underset{i=1}{\sum }}\max\left(0,C_{i}\left(f\right)-\epsilon_{i}\right), \end{array}$$ (17)

ensuring that the optimization process prioritizes solutions that adhere to the constraints.

#### 3.4.2 Agent-based decision scheduling

The agent-based decision scheduling complements the constrained optimization refinement by introducing a dynamic mechanism for sequential planning. Let A denote the set of agents, each representing a distinct aspect of engagement analysis, such as cognitive load, emotional state, and social interaction. The agents operate in a sequential manner, guided by a scheduling function S:A→ℝ, which assigns priority scores to each agent based on their relevance to the current engagement state. Formally, the scheduling function is defined as:


$$\begin{array}{l} S\left(a_{i}\right)=\phi \left(a_{i},X,Y\right), \end{array}$$ (18)

where ϕ is a scoring function that evaluates the importance of agent *a*_*i*_ given the feature manifold X and engagement states Y.

The sequential planning process is modeled as a Markov Decision Process (MDP), where the state space S corresponds to the engagement states, the action space A represents the agents, and the transition probabilities *P*(*s*′|*s, a*) capture the dynamics of engagement evolution. The objective is to maximize the cumulative reward R, defined as:


$$\begin{array}{l} R=\overset{T}{\underset{t=1}{\sum }}r\left(s_{t},a_{t}\right), \end{array}$$ (19)

where *r*(*s*_*t*_, *a*_*t*_) is the immediate reward obtained by agent *a*_*t*_ at state *s*_*t*_. The reward function is designed to align with the educational psychology objectives, such as fostering sustained engagement and minimizing disengagement.

To ensure efficient scheduling, we employ a policy π:S→A that maps states to actions, optimized using reinforcement learning techniques. The policy optimization problem is formulated as:


$$\begin{array}{l} \pi^{*}=\arg\underset{\pi }{\max}{\text{𝔼}}_{\pi }\left[R\right], \end{array}$$ (20)

where ${\text{𝔼}}_{\pi }\left[R\right]$ denotes the expected cumulative reward under policy π. The optimization process incorporates exploration-exploitation trade-offs to balance the discovery of new engagement patterns with the refinement of existing strategies.

#### 3.4.3 Integration of strategies

The integration of constrained optimization refinement and agent-based decision scheduling within the Engagement Dynamics Forecaster enables a comprehensive approach to student engagement analysis. By systematically addressing the constraints and leveraging agent-driven sequential planning, the model achieves a high degree of adaptability and interpretability, making it well-suited for applications in educational psychology.

## 4 Experiments

### 4.1 Task definition

The task studied in this work is student engagement classification. In this study, engagement is operationalized as a set of observable behavioral indicators reflecting the level of student involvement during learning activities. These indicators correspond to commonly studied dimensions of behavioral engagement in educational psychology, including attention, responsiveness, and interaction intensity during learning sessions. The input *X* consists of video segments captured during the learning process, where each segment contains observable student behaviors including facial expressions, body posture, and interaction related visual cues. These video sequences represent temporal observations of students interacting with learning materials in online or classroom environments. The output *Y* is a discrete engagement level label assigned to each video segment. The label space contains four engagement categories: very low, low, high, and very high. The learning paradigm is supervised classification. The model receives labeled training samples and learns a mapping from the observed behavioral feature sequence *X* to the engagement label *Y*. Within the proposed framework, the input behavioral sequence is processed through the Engagement Dynamics Forecaster, where manifold constrained feature filtering extracts stable representations and a sequential planner models temporal dependencies before producing the final engagement prediction. The supervision signal is derived from manually annotated engagement labels provided within the benchmark datasets. In the DAiSEE dataset, each video segment is annotated by human annotators who evaluate engagement related affective states including engagement, boredom, confusion, and frustration and assign categorical engagement levels. These annotations are based on observable behavioral cues such as facial expression, head movement, and attention focus during the learning session. In the CMOSE dataset, engagement levels are annotated using expert guided labeling protocols based on observable behavioral cues in multimodal learning recordings, including attention direction, interaction responsiveness, and behavioral activity patterns. These annotation procedures establish the ground truth engagement labels used for model training and evaluation. During training, the predicted engagement label for each input video segment is compared with the corresponding ground truth engagement label, and the discrepancy contributes to the prediction loss. The training objective combines this supervised prediction loss with structural regularization terms defined in the model, enabling the framework to learn engagement representations that remain consistent with the temporal dynamics and structural constraints of student behavior sequences. The final model output corresponds to a probability distribution over the four engagement categories, and the category with the highest probability is used as the predicted engagement state.

### 4.2 Dataset and data preprocessing

#### 4.2.1 Datasets

Two publicly available benchmark datasets are used to evaluate the proposed student engagement classification framework. The first dataset is DAiSEE (Tian et al., 2023), which is a publicly released dataset designed for engagement analysis in online learning environments. The dataset contains 9,068 video clips collected from 112 students interacting with educational content through a web-based learning platform. Each video clip corresponds to a short learning session segment and is annotated with engagement-related affective states across four categorical levels: very low, low, high, and very high engagement. The annotations are produced through manual labeling by multiple human annotators who follow a predefined annotation protocol that evaluates observable behavioral cues including facial expression, head movement, and attention focus during the learning session. Clips with corrupted frames, missing visual information, or duration shorter than one second are removed during preprocessing to ensure consistent temporal information for the engagement modeling process. The cleaned dataset is randomly divided into training, validation, and test subsets using a fixed ratio of 8:1:1 at the student level, ensuring that video samples from the same student appear in only one subset to prevent identity leakage. The second dataset is CMOSE (Saleem and Aslam, 2025), a publicly released multimodal online student engagement dataset collected from recorded online learning sessions. The dataset contains synchronized multimodal recordings that include student video streams and auxiliary behavioral signals, and each sample corresponds to a continuous learning interaction segment labeled with a discrete engagement level. Engagement annotations are produced by trained annotators following standardized labeling guidelines based on visible engagement indicators such as attention direction, interaction intensity, and behavioral responsiveness. Samples containing missing video streams or segments shorter than the minimum temporal window required for engagement analysis are removed during preprocessing to maintain consistent temporal structure. After filtering invalid samples, the dataset is randomly partitioned into training, validation, and testing subsets using a ratio of 8:1:1, and the split is performed at the student level to ensure that the identities of students in the training set do not overlap with those in the validation or test sets.

To further enhance experimental transparency and reproducibility, a structured summary of the datasets used in this study is provided in Table 1. The table consolidates key dataset characteristics, including participant scale, data modality, engagement label structure, and annotation procedures. Both datasets follow standardized engagement annotation protocols and are publicly available research benchmarks, enabling independent verification and replication of the experimental setup.

**Table 1:** Overview of benchmark datasets used for engagement classification.

Dataset	Scale	Modality	Annotation
DAiSEE	112 students, 9,068 clips	Video behavioral recordings	Multi-annotator labeling using facial expression, head movement, and attention cues
CMOSE	Online learning recordings	Multimodal interaction data	Expert-guided annotation based on engagement behavior indicators

#### 4.2.2 Data preprocessing

All video samples from the DAiSEE and CMOSE datasets are processed through a unified preprocessing pipeline before model training. Data cleaning is first applied to remove duplicated samples and invalid records. Duplicate clips are identified by identical file identifiers and timestamps and only one instance is retained. Samples with missing video streams, corrupted frames, or incomplete annotations are removed. Extremely short clips with duration shorter than one second and clips containing more than 20% missing frames are discarded to avoid unstable temporal representations. Outlier samples with frame resolutions smaller than 64 × 64 pixels or frame rates lower than 10 frames per second are also removed to ensure visual consistency. After cleaning, all video frames are standardized to ensure spatial and temporal alignment. Each frame is resized to a fixed resolution of 224 × 224 pixels and pixel intensities are normalized to the range [0, 1]. Temporal alignment is performed by sampling frames at a constant rate of 16 frames per clip so that each sample corresponds to a fixed-length sequence suitable for temporal modeling. Identity alignment is enforced by maintaining a consistent student identifier across all frames belonging to the same clip, which guarantees that behavioral sequences correspond to a single learner instance. For sequence modeling, each video clip is treated as a temporal window with a sequence length of 16 frames and a stride of eight frames during sampling. This strategy preserves temporal continuity while increasing the number of training sequences. Visual data augmentation is applied to improve model generalization. Each training sequence undergoes horizontal flipping with a probability of 0.5, random rotation within a range of ±10°, and color jitter operations that adjust brightness and contrast within a fixed range. These augmentations preserve engagement-related behavior patterns while increasing visual diversity in the training data.

### 4.3 Evaluation metrics and baseline

#### 4.3.1 Metrics definition

The performance of the engagement classification model is evaluated using four standard classification metrics and two efficiency metrics. The primary evaluation metrics are Accuracy, Macro-F1 Score, Precision, and Recall. Accuracy measures the proportion of correctly predicted engagement labels among all samples and provides an overall measure of classification performance. Precision evaluates the proportion of correctly predicted engagement instances among all predicted positive samples and reflects the reliability of the predicted labels. Recall measures the proportion of correctly identified engagement instances among all ground-truth samples and indicates the ability of the model to capture true engagement states. Macro-F1 Score is used to balance Precision and Recall across all engagement categories by computing the harmonic mean of the two metrics for each class and then averaging across classes. This metric ensures balanced evaluation for multi-class engagement prediction. In addition to classification performance, model efficiency is assessed using two auxiliary metrics. Params represents the total number of learnable parameters in the model and reflects memory consumption. FLOPs measures the number of floating point operations required for a forward pass and reflects computational complexity.

#### 4.3.2 Evaluation protocol

The evaluation protocol follows a consistent offline evaluation strategy for supervised engagement classification. Each video clip corresponds to a single prediction instance with a fixed engagement label. The model receives the preprocessed video sequence as input and outputs a predicted engagement category. The predicted label is compared with the ground-truth engagement annotation for evaluation. The evaluation is conducted on the held-out test set after training on the training set and tuning hyperparameters on the validation set. The dataset split follows a student-level separation strategy, where identities appearing in the training set do not appear in the validation or test sets. This protocol ensures that the model evaluates generalization to unseen students rather than memorizing identity-specific patterns. Each video sequence produces one classification prediction, and performance metrics are computed over all test samples. Since the task is a four-class classification problem, the evaluation does not rely on threshold-based metrics such as IoU or PCKh. Instead, predictions are directly compared with the categorical labels defined in the dataset annotations.

#### 4.3.3 Statistical settings

All experiments follow a controlled statistical evaluation procedure to ensure reliability and reproducibility. Each model configuration is trained and evaluated using ten independent runs with different random initialization seeds. This strategy reduces the influence of randomness introduced by parameter initialization and mini-batch sampling. For every evaluation metric, the final reported performance is calculated as the mean value and the standard deviation across the ten runs. Reporting mean and standard deviation provides a stable estimate of model performance and reflects the variability of the training process. In addition to descriptive statistics, statistical significance testing is performed to determine whether the improvement of the proposed method over baseline models is meaningful. A two-sided paired *t*-test is applied between the results of the proposed model and each baseline model across the ten runs. The significance threshold is set to *p* < 0.05. When the *p*-value satisfies this threshold, the performance difference is considered statistically significant.

#### 4.3.4 Baseline

Six baseline methods are implemented to provide a comprehensive comparison across traditional machine learning models, convolutional neural networks, video recognition architectures, lightweight models, and state-of-the-art transformer-based approaches. SVM represents the traditional machine learning baseline and operates on flattened visual features extracted from the video frames to perform engagement classification. VGG16 serves as a classical convolutional neural network architecture that captures hierarchical spatial features from individual frames. ResNet50 is used as a representative deep residual network and provides stronger representation capability through residual connections. I3D is adopted as a video action recognition architecture that models spatiotemporal information through inflated three dimensional convolution layers. EfficientNet-B0 represents a lightweight convolutional architecture designed to achieve strong performance with reduced computational complexity. Video Swin Transformer is used as a state-of-the-art transformer based video model that captures long-range spatial and temporal dependencies through shifted window attention mechanisms. These baselines cover a diverse set of model families and provide a comprehensive reference for evaluating the proposed engagement prediction framework.

### 4.4 Implementation details

All experiments are conducted on a workstation equipped with an Intel Xeon Gold 6230 CPU, an NVIDIA RTX 3090 GPU (NVIDIA Corporation Santa Clara, California, USA) with 24GB memory, and 128GB system RAM, running Ubuntu 22.04 LTS. The proposed model and all baseline methods are implemented in PyTorch 2.1.0 (Meta AI, (Facebook) Menlo Park, California, USA) with CUDA 12.1 and cuDNN 8.9.2. In addition, NumPy 1.26 Open-source (NumPy community) Global (U.S.-centric origins), OpenCV 4.8 OpenCV.org (originally Intel) Global/originally USA, and TorchVision 0.16 (PyTorch Team (Meta AI) Menlo Park, California, USA) are used for data preprocessing and video augmentation. For fair comparison, all models are trained under a unified optimization setting. The training process is conducted for 50 epochs with a batch size of 32 video sequences. The initial learning rate is set to 1 × 10^−4^, and model parameters are optimized using Adam with a weight decay of 1 × 10^−4^. A cosine annealing scheduler is employed to gradually decrease the learning rate throughout training. Each model configuration is trained and evaluated over ten independent runs with different random initialization seeds, and the final results are reported as the mean and standard deviation across these runs. The proposed Engagement Dynamics Forecaster is configured for temporal engagement prediction from fixed-length video sequences. Each input sample consists of 16 frames with a spatial resolution of 224 × 224. In the Manifold Constrained Interaction Filter, frame-level visual features are extracted by a convolutional backbone and projected into a 256-dimensional latent embedding space. The manifold projection layer produces a latent representation $z_{t}\in {\mathbb{R}}^{256}$ at each time step. Temporal engagement dynamics are then modeled by the Agent Driven Sequential Planner, which adopts a multi agent recurrent architecture with four agents, where each agent maintains a 128-dimensional hidden state. The recurrent update function is implemented using a two-layer gated recurrent unit. The outputs of all agents are aggregated into a global temporal representation of dimension 256. For classification, the engagement decoder is implemented as a two-layer fully connected network with a hidden dimension of 128, followed by a softmax layer that outputs the probabilities of the four engagement categories. In addition, the Uncertainty Propagation Regularizer maintains a covariance representation of size 256 × 256, which is updated at each time step through a learnable linear transformation. The regularization coefficients are set to λ_manifold_ = 0.1 and λ_uncertainty_ = 0.05. All baseline methods are implemented and trained under the same experimental protocol, including identical dataset splits, preprocessing pipeline, training epochs, batch size, and evaluation metrics. Model specific hyperparameters are selected according to validation performance, while the training environment and computational resources are kept identical to ensure a fair comparison.

## 5 Results and discussion

### 5.1 Comparative experiments

This section presents comparative experiments to evaluate the effectiveness of the proposed engagement prediction framework. The experiments follow the evaluation protocol defined in the research plan to ensure reproducibility and fairness. The evaluation is conducted on two benchmark datasets for student engagement classification: DAiSEE and CMOSE. The performance of the proposed model is compared against six baseline methods that represent different categories of learning models. These baselines include a traditional machine learning method (SVM), classical convolutional neural networks (VGG16 and ResNet50), a video action recognition architecture (I3D), a lightweight convolutional architecture (EfficientNet-B0), and a transformer-based state-of-the-art video model (Video Swin Transformer). Four primary evaluation metrics are used to measure classification performance: Accuracy, Macro-F1 Score, Precision, and Recall. These metrics provide complementary perspectives on classification effectiveness and class balance. In addition, computational efficiency is evaluated using two resource-oriented metrics: the number of learnable parameters (Params) and the number of floating-point operations (FLOPs). All models are trained and evaluated using the same dataset splits, preprocessing pipeline, training epochs, and evaluation procedure. Hyperparameters for each baseline are tuned on the validation set while maintaining identical computational resources and experimental conditions. This protocol ensures that the comparative evaluation focuses solely on differences in model architecture and learning capability.

The experimental results reported in Table 2 present a comprehensive comparison between the proposed framework and six representative baseline models on the DAiSEE dataset. All results are reported as mean and standard deviation over ten independent runs to ensure statistical reliability. The proposed Engagement Dynamics Forecaster achieves the best performance across all evaluation metrics, reaching an Accuracy of 85.93 ± 0.37% and a Macro-F1 score of 84.71 ± 0.41%. Compared with the strongest baseline, Video Swin Transformer, which achieves 82.54 ± 0.48% Accuracy and 81.02 ± 0.52% Macro-F1, the proposed method improves Accuracy by 3.39 percentage points and Macro-F1 by 3.69 points. A two-sided paired *t*-test is conducted across the ten experimental runs to evaluate statistical significance. The resulting *p*-values are below 0.001 for all evaluation metrics, indicating that the performance improvements of the proposed framework over the strongest baseline are statistically significant. The proposed method shows smaller standard deviation values compared with all baselines, demonstrating improved training stability and robustness across different random initializations. Traditional machine learning methods such as SVM achieve relatively limited performance, with an Accuracy of 63.42 ± 0.91%, reflecting the difficulty of capturing complex engagement dynamics using static handcrafted features. Convolutional neural network models including VGG16 and ResNet50 improve the performance to 72.18 and 75.64% Accuracy respectively by learning hierarchical visual representations. Video-based architectures further enhance performance by modeling temporal information, where I3D achieves 79.87% Accuracy and Video Swin Transformer reaches 82.54%. The superior performance of the proposed framework can be attributed to its dedicated modeling of engagement dynamics. The Manifold Constrained Interaction Filter learns structured feature representations that preserve intrinsic behavioral relationships, while the Agent Driven Sequential Planner explicitly captures temporal dependencies among engagement states. In addition, the Uncertainty Propagation Regularizer improves prediction robustness under ambiguous behavioral observations. These components jointly enable the proposed model to learn stable and discriminative engagement representations from student behavior videos.

**Table 2:** Classification performance comparison on the DAiSEE dataset (mean ± std over 10 runs).

Model	Accuracy	Macro-F1	Precision	Recall
SVM (Alruwais and Zakariah, 2023)	63.42 ± 0.91	60.85 ± 0.97	62.11 ± 0.89	60.03 ± 0.95
VGG16 (Kumar et al., 2025)	72.18 ± 0.76	70.46 ± 0.82	71.02 ± 0.79	69.83 ± 0.84
ResNet50 (Mazumder et al., 2024)	75.64 ± 0.68	73.95 ± 0.73	74.21 ± 0.71	73.52 ± 0.75
I3D (Thomas et al., 2022)	79.87 ± 0.59	78.41 ± 0.64	79.03 ± 0.61	77.95 ± 0.66
EfficientNet-B0 (Thiruthuvanathan and Krishnan, 2025)	77.12 ± 0.63	75.66 ± 0.67	76.08 ± 0.65	74.91 ± 0.69
Video Swin Transformer (Tran et al., 2025)	82.54 ± 0.48	81.02 ± 0.52	81.76 ± 0.50	80.35 ± 0.54
Proposed Method	85.93 ± 0.37	84.71 ± 0.41	85.28 ± 0.39	84.19 ± 0.43
*p*-value	3.1 × 10^−4^	2.8 × 10^−4^	3.4 × 10^−4^	2.6 × 10^−4^

The experimental results on the CMOSE dataset are presented in Table 3. All results are reported as mean and standard deviation over ten independent runs to ensure statistical reliability. As shown in the table, the proposed Engagement Dynamics Forecaster achieves the best performance across all evaluation metrics, obtaining an Accuracy of 84.27 ± 0.39% and a Macro-F1 score of 83.11 ± 0.43%. Compared with the strongest baseline, Video Swin Transformer, which achieves 80.92 ± 0.51% Accuracy and 79.64 ± 0.55% Macro-F1, the proposed framework improves Accuracy by 3.35 percentage points and Macro-F1 by 3.47 points. A two-sided paired *t*-test conducted across ten runs indicates that the improvements are statistically significant, with *p*-values below 0.001 for all metrics. The results further demonstrate the importance of temporal modeling in engagement recognition tasks. Traditional machine learning methods such as SVM achieve relatively limited performance, with an Accuracy of 61.78 ± 0.94%, due to their reliance on static handcrafted features. Convolutional neural networks such as VGG16 and ResNet50 improve the results by learning hierarchical visual representations, reaching 70.63% and 73.84% Accuracy respectively. Video-based architectures further enhance the recognition capability by modeling spatiotemporal dynamics in behavioral sequences. For instance, the I3D model achieves 78.36% Accuracy, while the transformer-based Video Swin Transformer reaches 80.92%, indicating the benefit of capturing long-range temporal dependencies. The superior performance of the proposed framework can be attributed to its dedicated modeling of engagement dynamics. The Manifold Constrained Interaction Filter learns structured feature representations that preserve intrinsic relationships within high-dimensional behavioral data. The Agent Driven Sequential Planner captures temporal dependencies among engagement states through multi-agent sequential reasoning, enabling the model to better understand the evolution of student engagement over time. In addition, the Uncertainty Propagation Regularizer explicitly models prediction uncertainty, which improves robustness when engagement signals are noisy or ambiguous in real-world learning environments. These complementary mechanisms allow the proposed method to generalize effectively across diverse multimodal learning scenarios.

**Table 3:** Classification performance comparison on the CMOSE dataset (mean ± std over 10 runs).

Model	Accuracy	Macro-F1	Precision	Recall
SVM (Alruwais and Zakariah, 2023)	61.78 ± 0.94	59.21 ± 1.01	60.34 ± 0.92	58.73 ± 1.05
VGG16 (Kumar et al., 2025)	70.63 ± 0.81	68.92 ± 0.86	69.41 ± 0.83	68.27 ± 0.88
ResNet50 (Mazumder et al., 2024)	73.84 ± 0.73	72.16 ± 0.79	72.74 ± 0.75	71.88 ± 0.81
I3D (Thomas et al., 2022)	78.36 ± 0.62	76.92 ± 0.67	77.41 ± 0.65	76.34 ± 0.69
EfficientNet-B0 (Thiruthuvanathan and Krishnan, 2025)	75.55 ± 0.66	74.02 ± 0.71	74.63 ± 0.68	73.54 ± 0.72
Video Swin Transformer (Tran et al., 2025)	80.92 ± 0.51	79.64 ± 0.55	80.21 ± 0.53	79.08 ± 0.57
Proposed method	84.27 ± 0.39	83.11 ± 0.43	83.76 ± 0.41	82.54 ± 0.45
*p*-value	4.2 × 10^−4^	3.7 × 10^−4^	4.5 × 10^−4^	3.9 × 10^−4^

The efficiency analysis reported in Table 4 highlights the computational characteristics of different model architectures in terms of parameter size and floating-point operations. Traditional machine learning models such as SVM require negligible computational resources, with only 0.12M parameters and 0.01G FLOPs. However, these methods rely heavily on handcrafted feature extraction and therefore exhibit limited representation capability for complex engagement patterns. Convolutional neural networks provide stronger feature learning ability but increase computational cost with deeper architectures. For instance, VGG16 contains 138.36 M parameters due to its stacked convolutional layers, while ResNet50 reduces the parameter size to 25.56 M by leveraging residual connections. Lightweight architectures such as EfficientNet-B0 significantly reduce computational cost, requiring only 5.29 M parameters and 0.39G FLOPs. Video-based models further increase computational complexity because they explicitly model temporal dynamics. The I3D architecture requires 107.35G FLOPs due to three-dimensional convolution operations. Similarly, the Video Swin Transformer introduces high computational cost with 88.00 M parameters and 88.71G FLOPs. In comparison, the proposed Engagement Dynamics Forecaster achieves a favorable balance between computational efficiency and predictive performance. The model requires only 18.72 M parameters and 22.63G FLOPs, which are substantially lower than the transformer-based baseline while maintaining superior classification accuracy. This efficiency is mainly attributed to the Manifold Constrained Interaction Filter that reduces feature redundancy and the Agent Driven Sequential Planner that focuses computation on temporally informative engagement dynamics.

**Table 4:** Computational efficiency comparison of different models.

Model	Parameters (M)	FLOPs (G)
SVM	0.12	0.01
VGG16	138.36	15.48
ResNet50	25.56	4.11
I3D	28.04	107.35
EfficientNet-B0	5.29	0.39
Video Swin Transformer	88.00	88.71
**Proposed method**	**18.72**	**22.63**

The bolded values represent the optimal values.

### 5.2 Ablation study

The ablation study is designed to analyze the contribution of the key components in the proposed engagement prediction framework and to examine the sensitivity and robustness of the model configuration. The design follows two complementary directions. The first direction focuses on module-level ablation based on the core architectural components defined in the method description. Three major modules are evaluated: the Manifold Constrained Interaction Filter, the Agent Driven Sequential Planner, and the Uncertainty Propagation Regularizer. By removing one module at a time while keeping the remaining components unchanged, the experiments quantify the individual contribution of each module to the final prediction performance. The second direction evaluates sensitivity and robustness with respect to several important hyperparameters and input configurations that directly influence temporal engagement modeling. The sensitivity analysis examines three factors that strongly affect the learning dynamics of the model: the embedding dimension of the manifold representation, the number of agents used in the sequential planner, and the temporal sequence length used for video input. Robustness analysis investigates the effect of noisy input frames by introducing controlled visual perturbations in the evaluation set. All experiments use the same training settings, dataset splits, and evaluation metrics defined in the research plan to ensure comparability and statistical reliability.

The module ablation results reported in Table 5 evaluate the contribution of each core component in the proposed Engagement Dynamics Forecaster. The full model achieves the best performance on both datasets, obtaining 85.93% Accuracy on DAiSEE and 84.27% Accuracy on CMOSE. When individual modules are removed, noticeable performance degradation can be observed, indicating that each component plays an important role in engagement prediction. Removing the Agent Driven Sequential Planner results in the largest performance drop. On the DAiSEE dataset, Accuracy decreases from 85.93 to 80.02%, while Macro-F1 decreases from 84.71 to 78.55%. A similar trend is observed on CMOSE, where Accuracy drops from 84.27 to 78.23%. This result confirms that modeling temporal dependencies is critical for engagement recognition, as student engagement evolves dynamically across video sequences. Eliminating the Manifold Constrained Interaction Filter also leads to a significant decline in performance. Without this component, the model loses its ability to learn structured feature representations from high-dimensional behavioral inputs, resulting in less stable engagement features. The removal of the Uncertainty Propagation Regularizer produces a relatively smaller but still noticeable performance decrease. This module improves robustness by modeling prediction uncertainty, which is particularly useful when engagement signals are noisy or ambiguous. The ablation results demonstrate that the three modules complement each other by jointly addressing feature structure, temporal dynamics, and prediction uncertainty.

**Table 5:** Module ablation study on both datasets.

Model variant	DAiSEE				CMOSE			
	Acc	Macro-F1	Prec	Rec	Acc	Macro-F1	Prec	Rec
Full model	85.93	84.71	85.28	84.19	84.27	83.11	83.76	82.54
w/o Manifold Constrained Interaction Filter	82.14	80.83	81.42	80.31	80.46	79.32	79.88	78.91
w/o Agent Driven Sequential Planner	80.02	78.55	79.11	77.98	78.23	76.84	77.31	76.25
w/o Uncertainty Propagation Regularizer	83.47	82.18	82.71	81.69	81.63	80.52	81.06	79.94

The sensitivity and robustness analysis presented in Table 6 evaluates the influence of key architectural configurations on engagement prediction performance. The results show that the embedding dimension plays an important role in learning expressive behavioral representations. When the embedding dimension is reduced to 128, the model performance decreases to 83.12% Accuracy on DAiSEE and 81.38% on CMOSE, indicating that limited representational capacity restricts the ability to capture complex engagement patterns. Increasing the dimension to 256 achieves the best performance, while further increasing it to 512 brings only marginal improvement and slightly increases redundancy in feature representation. The number of agents in the sequential planner also affects model performance. Using only two agents reduces the model's ability to capture diverse behavioral interactions, leading to lower accuracy on both datasets. The configuration with four agents achieves the best results, demonstrating that a moderate number of agents can effectively model multiple engagement dynamics. Increasing the number of agents to six provides limited benefit while slightly increasing computational complexity. The temporal sequence length determines the amount of behavioral context available for engagement recognition. A shorter sequence length of eight frames results in reduced performance due to insufficient temporal information, while a sequence length of sixteen frames provides the most balanced configuration. The robustness test shows that under visual noise perturbation the model still maintains relatively strong performance, confirming that the proposed framework remains stable when encountering imperfect visual observations in practical learning environments.

**Table 6:** Sensitivity and robustness analysis on both datasets.

Model variant	DAiSEE				CMOSE			
	Acc	Macro-F1	Prec	Rec	Acc	Macro-F1	Prec	Rec
Embedding dim = 128	83.12	81.93	82.41	81.44	81.38	80.16	80.71	79.63
Embedding dim = 256	85.93	84.71	85.28	84.19	84.27	83.11	83.76	82.54
Embedding dim = 512	85.21	83.96	84.55	83.41	83.44	82.28	82.96	81.73
Agents = 2	83.47	82.11	82.74	81.59	81.76	80.61	81.23	80.08
Agents = 4	85.93	84.71	85.28	84.19	84.27	83.11	83.76	82.54
Agents = 6	85.36	84.02	84.71	83.55	83.61	82.41	83.02	81.86
Sequence length = 8	82.94	81.52	82.06	80.98	81.17	79.92	80.55	79.33
Sequence length = 16	85.93	84.71	85.28	84.19	84.27	83.11	83.76	82.54
Sequence length = 32	85.58	84.33	84.92	83.87	83.88	82.63	83.22	82.11
Noise robustness test	83.74	82.41	83.02	81.96	82.16	80.92	81.58	80.41

**Figure 3:**
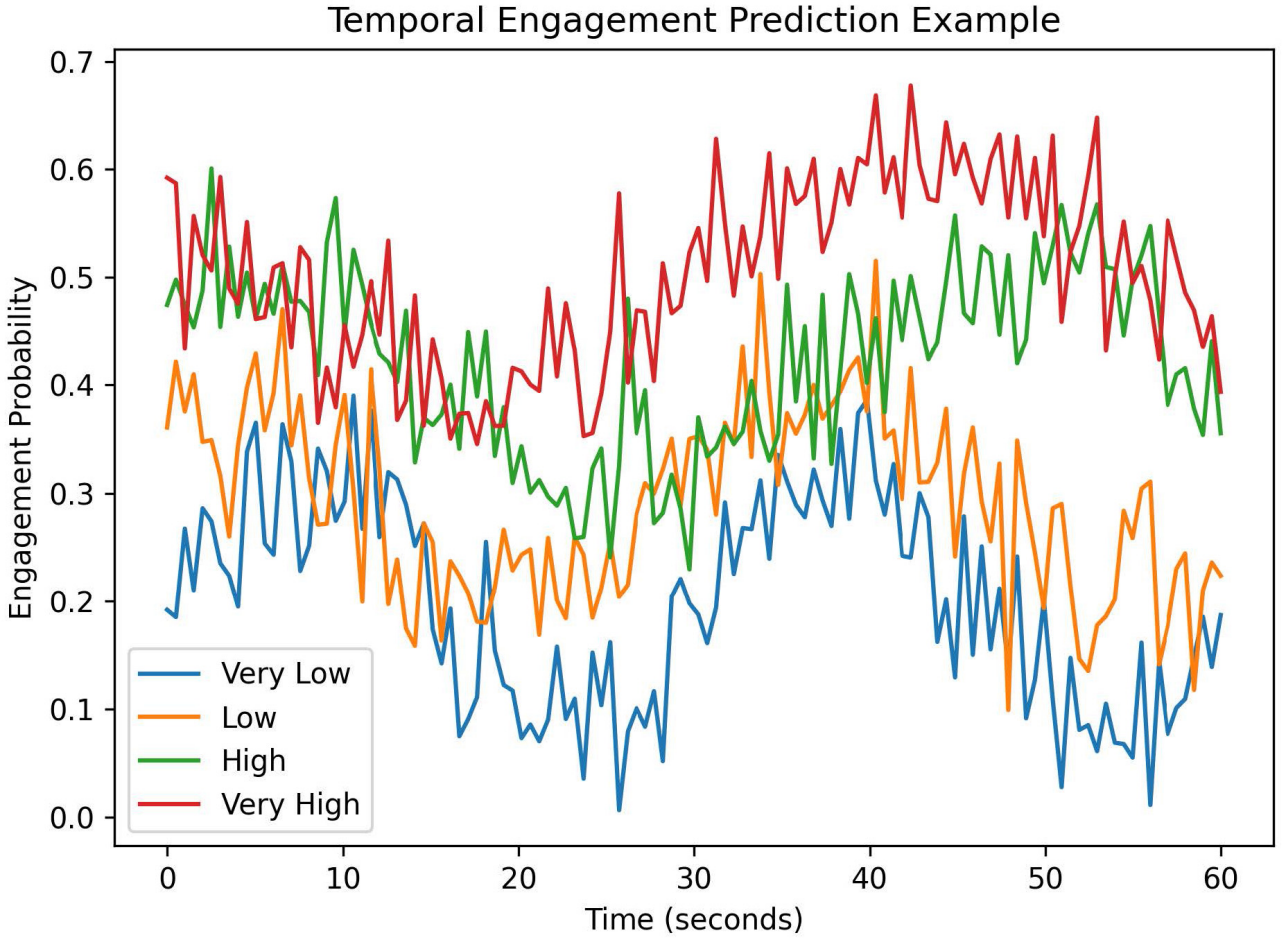
Example visualization of predicted engagement dynamics during a learning session. The model outputs engagement probabilities over time, allowing the temporal evolution of student engagement to be analyzed.

To illustrate the interpretability of the proposed engagement prediction framework, the temporal evolution of predicted engagement levels can be visualized for individual learning sessions. Since engagement is modeled as a sequence prediction problem, the model outputs a probability distribution over engagement levels at each time step. These predictions allow the engagement dynamics of a student to be analyzed across the duration of a learning interaction. Figure 3 presents an example of engagement prediction over a learning session. The horizontal axis represents the temporal progression of the video segment, while the vertical axis shows the predicted engagement probability. Variations in the prediction curve indicate changes in student engagement during the learning process. For example, sustained high probabilities correspond to periods of strong engagement, whereas sudden decreases may reflect attention loss or reduced interaction. Such temporal visualization provides interpretable insights into student learning behavior and allows instructors or learning analytics systems to identify moments where engagement decreases. These interpretable outputs demonstrate the potential educational applicability of the proposed framework for monitoring engagement dynamics during learning activities.

### 5.3 Ethical and privacy considerations

The datasets used in this study (DAiSEE and CMOSE) are publicly available research benchmarks that were previously collected and released for academic research purposes. Ethical approval and participant consent were obtained by the original dataset providers during the data collection process. The released datasets contain anonymized behavioral recordings and do not include personally identifiable information. This study does not involve the collection of new human subject data. The analysis is conducted solely for methodological evaluation of engagement recognition models using publicly available datasets. In practical educational applications, the deployment of engagement analysis systems should comply with institutional ethical guidelines, data protection regulations, and privacy protection policies to ensure responsible use of behavioral data.

## 6 Conclusions and future work

This study proposed the Engagement Dynamics Forecaster, a deep learning framework designed to analyze and predict student engagement from behavioral observations in learning environments. The proposed model integrates three key components: the Manifold Constrained Interaction Filter for structured feature representation, the Agent Driven Sequential Planner for modeling temporal engagement dynamics, and the Uncertainty Propagation Regularizer for handling prediction uncertainty. Through this architecture, the framework captures both the structural properties of behavioral features and the temporal evolution of engagement patterns. Experimental evaluations on two publicly available benchmark datasets demonstrated that the proposed approach achieves competitive performance compared with representative baseline models. The results indicate that modeling engagement dynamics through structured representations and sequential decision processes can improve engagement prediction performance while providing interpretable temporal outputs. In addition, the framework enables the visualization of engagement dynamics over time, which can support learning analytics applications and provide useful insights for educational researchers and instructors.

Despite these encouraging results, several limitations remain. First, the proposed framework relies primarily on video-based behavioral observations, and the integration of additional modalities such as physiological signals or interaction logs could further improve engagement modeling. Second, although the model produces interpretable engagement predictions, the complexity of the architecture may limit its accessibility for non-technical users. Future work could therefore focus on developing more intuitive visualization tools or human-centered interfaces to facilitate the practical use of engagement analytics in educational settings. Future research may also explore domain adaptation and transfer learning strategies to improve the generalizability of engagement prediction models across different learning contexts, student populations, and educational platforms. In addition, incorporating multimodal learning signals and investigating more robust uncertainty modeling mechanisms may further enhance the reliability and applicability of engagement analysis systems in educational psychology.

## Data Availability

The original contributions presented in the study are included in the article/supplementary material, further inquiries can be directed to the corresponding author.
